# An Early Myeloma Bone Disease Model in Skeletally Mature Mice as a Platform for Biomaterial Characterization of the Extracellular Matrix

**DOI:** 10.1155/2020/3985315

**Published:** 2020-06-27

**Authors:** Fani Ziouti, Ana Prates Soares, Inés Moreno-Jiménez, Alexander Rack, Bjarne Bogen, Amaia Cipitria, Paul Zaslansky, Franziska Jundt

**Affiliations:** ^1^Department of Internal Medicine II, University Hospital Würzburg, Oberdürrbacher Straße 6, 97080 Würzburg, Germany; ^2^Department for Operative and Preventive Dentistry, Centrum für Zahn-, Mund- und Kieferheilkunde, Charité—Universitätsmedizin Berlin, Aßmannshauser Str. 4-6, 14197 Berlin, Germany; ^3^Max Planck Institute of Colloids and Interfaces, Department of Biomaterials, Am Mühlenberg 1, 14476 Potsdam, Germany; ^4^ESRF—The European Synchrotron, 71 Avenue des Martyrs, 38000 Grenoble, France; ^5^Department of Immunology, Institute of Clinical Medicine, University of Oslo and Oslo University Hospital, Sognsvannsveien 20, 0372 Oslo, Norway; ^6^Comprehensive Cancer Center Mainfranken, Josef-Schneider Str. 6, 97080 Würzburg, Germany

## Abstract

Multiple myeloma (MM) bone disease is characterized by osteolytic bone tissue destruction resulting in bone pain, fractures, vertebral collapse, and spinal cord compression in patients. Upon initial diagnosis of MM, almost 80% of patients suffer from bone disease. Earlier diagnosis and intervention in MM bone disease would potentially improve treatment outcome and patient survival. New preclinical models are needed for developing novel diagnostic markers of bone structural changes as early as possible in the disease course. Here, we report a proof-of-concept, syngeneic, intrafemoral MOPC315.BM MM murine model in skeletally mature BALB/c mice for detection and characterization of very early changes in the extracellular matrix (ECM) of MM-injected animals. Bioluminescence imaging (BLI) in vivo confirmed myeloma engraftment in 100% of the animals with high osteoclast activity within 21 days after tumor cell inoculation. Early signs of aggressive bone turnover were observed on the outer bone surfaces by high-resolution microcomputed tomography (microCT). Synchrotron phase contrast-enhanced microcomputer tomography (PCE-CT) revealed very local microarchitecture differences highlighting numerous active sites of erosion and new bone at the micrometer scale. Correlative backscattered electron imaging (BSE) and confocal laser scanning microscopy allowed direct comparison of mineralized and nonmineralized matrix changes in the cortical bone. The osteocyte lacunar-canalicular network (OLCN) architecture was disorganized, and irregular-shaped osteocyte lacunae were observed in MM-injected bones after 21 days. Our model provides a potential platform to further evaluate pathological MM bone lesion development at the micro- and ultrastructural levels. These promising results make it possible to combine material science and pharmacological investigations that may improve early detection and treatment of MM bone disease.

## 1. Introduction

Among the patients diagnosed with multiple myeloma (MM), 80% already suffer from MM bone disease, exhibiting osteolytic bone tissue destruction or osteopenia, with symptoms of severe pain and fractures [1]. Morbidity and mortality due to MM bone disease are high, and quality of life is severely affected by skeletal-related pathologies [1, 2]. MM is rarely curable and is the second most common hematological neoplasia in the USA and Europe with an age-adjusted incidence of six per 100,000 people/year and a median age of 69 [3]. The pathological cells are clonal plasma cells in the bone marrow that secrete excessive amounts of monoclonal immunoglobulins. MM cells diffusely infiltrate the bone marrow or grow as multiple focal lesions. In advanced stages, extramedullary lesions develop. MM cells inhibit osteoblast differentiation and stimulate osteoclast function [4] resulting in increased bone resorption and characteristic osteolytic punched-out bone lesions as well as osteopenia. The current gold standard of treatment includes the use of bisphosphonates, local irradiation, and orthopedic intervention [1]. To date, MM treatment has not succeeded in healing bone lesions or regenerating bone tissue even in the absence of signs of active disease [5]. New approaches are urgently needed to lead to better detection and improved monitoring of bone structural changes as early as possible in the disease course, ideally before overt lytic lesions develop. This is critical for future improvements in diagnosis, novel treatment development, and for raising quality of life in MM patients, typically elderly patients.

Preclinical animal models are essential for the above and for developing novel diagnostic markers. They are also essential to better understand interactions between the extracellular matrix and tumor cells. Especially for human MM bone disease, reliable mouse models facilitate tracking lesion formation, expansion, and localization in the very early stages of the disease because they further help benchmark and quantify disease severity. We previously established the murine MOPC315.BM MM model [6]. Extensive osteolytic bone disease was demonstrated between 5 and 8 weeks after intravenous tumor cell injection in young 6-week-old BALB/c mice. Mice repetitively succumbed to paraplegia and extramedullary growth of MOPC315.BM.Luc cells [6].

Since MM is prevalent in aging populations, it is important to examine skeletally mature (adult) bones that differ substantially from young developing bones, where the mineralized tissue naturally undergoes extensive (re)modelling [7, 8]. Bone turnover, bone architecture, and adaptive bone formation/resorption substantially differ in bones of adult mice as compared with bones of young mice [9]. Higher bone formation and resorption activities in young (6 weeks old) compared to mature (16 weeks old) mice were linked to higher frequencies of growing osteolytic skeletal metastasis in in vivo models of breast and prostate cancer [10]. This however is very different in aging populations mostly suffering from MM disease, where normal bone turnover is very low. There is thus a great need for novel, syngeneic skeletally mature models of MM bone disease.

Here, we describe an intrafemoral (i.f.) murine MM model, where the disease is engrafted using MOPC315.BM.Luc cells [6, 11, 12]. This syngeneic model was created (i) to establish a controlled MM model in adult mice, (ii) to track disease development over time, and (iii) to confine MM disease primarily to one particular bone site. We further used the model (iv) to correlate MM engraftment by in vivo bioluminescence imaging (BLI) and (v) to facilitate high-resolution 3D mapping using a multiscale, multimodal material characterization approach.

## 2. Materials and Methods

### 2.1. Intrafemoral Injections and Bioluminescence Imaging (BLI)

26-week-old female BALB/c mice were obtained from Charles River (Sulzfeld, Germany). Local authorities approved all animal experiments (55.2 DMS-2532-2-31, Regierung of Unterfranken, Würzburg, Germany). The study was carried out in accordance with the replacement, reduction, refinement (3Rs) principle. BALB/c mice were either injected with PBS (*n* = 1) or with 10^5^ luciferase-positive MOPC315.BM.Luc cells (*n* = 10) [6], directly into the right femora between the condyles, through the patellar surface, into the bone marrow cavity. Mice were sacrificed on day 7 (*n* = 2), day 11 (*n* = 2), day 15 (*n* = 3), and day 21 (*n* = 3) after injection. BLI was performed in vivo to confirm MOPC315.BM.Luc cell engraftment and to monitor tumor progression over time as described previously [6]. Mice were sacrificed through cervical dislocation. The PBS control mouse was sacrificed at day 21. The severity of MM bone disease activity was quantified using Living Image 4.4 (PerkinElmer, Massachusetts, USA), and graphs were created with Prism 7 software (GraphPad, San Diego, USA). Femora were dissected, fixed in 4% paraformaldehyde for 48 hours, and transferred to 70% ethanol.

### 2.2. Tartrate-Resistant Acid Phosphatase (TRAP) Staining

For tartrate-resistant acid phosphatase (TRAP) staining, samples were dehydrated in increasing concentrations of ethanol, stained with 0.02 wt.% rhodamine 6G (AppliChem, St. Louis, USA), and prepared for cold embedding in polymethylmethacrylate (PMMA) (Technovit 9100, Heraeus Kulzer, Wehrheim, Germany). Nuclei counterstaining was achieved using Mayer's haematoxylin (MHS32, Sigma, Germany) applied for 20 seconds, washed in water, and blued in 0.1% ammonium hydroxide. A digital light microscope (VHX-S550E, Keyence, Neu-Isenburg, Germany) was used to image the stained sections.

### 2.3. Microcomputed Tomography (MicroCT) Imaging

For microcomputed tomography (microCT) imaging, samples were maintained in an ethanol atmosphere. Each bone was mounted in a 2 ml polyvinyl alcohol (PVA) vial (Sarstedt, Nümbrecht, Germany), centrally stabilized in styropore, and padded with a polyester foam saturated with 70% ethanol. MOPC315.BM.Luc samples were scanned using a Skyscan 1172 (Bruker microCT, Kontich, Belgium) with 2 *μ*m and 13.2 *μ*m effective pixel sizes, X-ray source set to 70 kV with a 1 mm thick aluminum filter, 2 s exposure time, and 1800 projections. Data were reconstructed (NRecon, Bruker microCT, Kontich, Belgium) and examined in 2D (ImageJ 1.52d, National Institutes of Health, Maryland, USA) and 3D (CTvox, Bruker microCT, Kontich, Belgium) to identify regions of high turnover and osteolytic lesions.

PMMA-embedded femora were scanned using an EasyTom 160 (RX Solutions, Chavanod, France). Scanning parameters were 45 kV, 45 *μ*A, 2.5 *μ*m voxel size, one frame per second, average frame of 8, and 1120 projections. Reconstruction of scan projections was performed using RX Solutions X-Act software.

### 2.4. Synchrotron Phase Contrast-Enhanced (PCE) MicroCT Imaging

Regions above the condyles extending ∼1.5 mm in length and covering the whole bone diameter were scanned on ID19 of the European Synchrotron Radiation Facility (ESRF, Grenoble, France). Synchrotron PCE-CT scans were recorded using the 34 keV harmonic in pink-beam mode and 650 nm effective pixel size, employing 45 mm propagation distance between the sample and detector (LSO scintillators and PCO edge camera). Typical scans required ∼5000 radiographic projections, with 300 ms exposure time, continuously rotating the samples by 360° using the so-called half acquisition mode. ESRF in-house code was used to reconstruct the data, enhancing contrast by means of Paganin-based filtering with a delta/beta ratio of 500 [13].

### 2.5. Backscattered Electron Imaging (BSE)

Backscattered electron (BSE) imaging was performed to investigate bone morphology and mineral content (brighter grey corresponds to higher mineral content) on regions with cortical lesions. Regions of interest were identified using high-resolution microCT. Controlled angle serial sectioning was performed to expose the PMMA block surface. BSE images were obtained using an environmental scanning electron microscope (FEI FEG-ESEM Quanta 600, FEI Company, Hillsboro, USA). This microscope operates at low vacuum (0.75 Torr), an accelerating voltage of 12.5 kV, a working distance of 9.9 mm, and a spot size of 4.0 [14]. Images taken at 100x magnification were merged together. Imaging conditions followed established quantitative backscattered electron imaging protocols for measurement of the mineral density distribution in human bone biopsies [15].

### 2.6. Fluorescence Confocal Laser Scanning Microscopy (CLSM)

Correlative imaging of the osteocyte lacunar-canalicular network (OLCN) was performed on the same exposed PMMA surface using a fluorescence confocal laser scanning microscope (CLSM, Leica TCS SP8 DLS Multiphoton, Wetzlar, Germany) [16]. The OLCN was visualized using *λ*_excitation_ = 514 nm/*λ*_emission_ = 550-650 nm laser light, magnification 40x, oil objective, 0.75 zoom, 6 tiles, 60 *μ*m total depth at 0.4 *μ*m step size. Multiple images were then merged.

For the network characterization, we used an established protocol based on previously published work [17, 18]. In short, raw CLSM data were segmented to automatically differentiate between canaliculi and lacunae based on their bulkiness. All datasets were then evaluated with the same segmentation parameter set. The average lacunae volume was calculated by counting segmented lacuna voxels and dividing by the total number of lacunae. The segmented canaliculi were then skeletonized and rendered into a 3D network for further quantitative analysis. Using this network, we then computed the canalicular density which quantifies the total length of canaliculi per unit bone volume, excluding lacunae volume.

## 3. Results

### 3.1. Intrafemoral Injections and Local Engraftment

**Figure 1:**
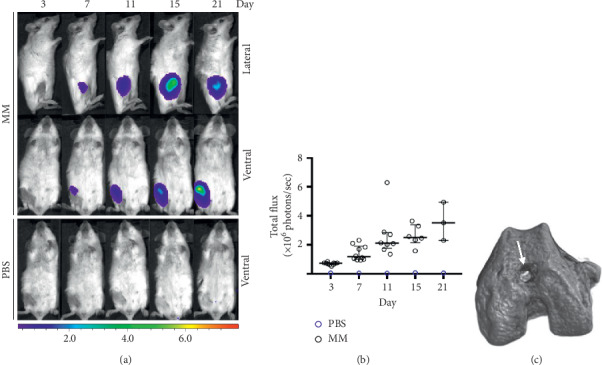
Detection of MOPC315.BM.Luc cells in 26-week-old BALB/c mice following inoculation. (a) BLI images of ventral and lateral views of a MOPC315.BM.Luc- and a PBS-injected mouse at increasing days after inoculation. (b) BLI signals (total flux in photons^−1^) of one control and ten MM mice at days after inoculation (median, interquartile range). (c) Typical femoral injection site (white arrow) in a 3D microCT reconstruction at day 21.

Inoculation of MOPC315.BM.Luc cells resulted in 100% engraftment in ten mice, as demonstrated in vivo by BLI (Figures 1(a) and 1(b)). Clear signs of established MOPC315.BM.Luc colonies were observed as early as day 7 (Figure 1(a)). BLI signals increased over the days following injection (Figure 1(b)) and were localized to the injected right femur (Figure 1(a)). All mice survived the entire experiment with no signs of suffering and the tumors appeared to steadily grow in the femora for 21 days during the experiment. All BLI signs were always restricted to the injected right femur (Figures 1(a) and 1(b)), though we cannot exclude the possibility that micrometastases reached other sites (e.g., liver). BLI signals have been shown to correlate with measurements of serum MOPC315.BM.Luc secreted immunoglobulin A levels and were therefore chosen for monitoring tumor growth in the MOPC315.BM model [6].

### 3.2. Detection of MOPC315.BM Cells and High Osteoclast Activity

**Figure 2:**
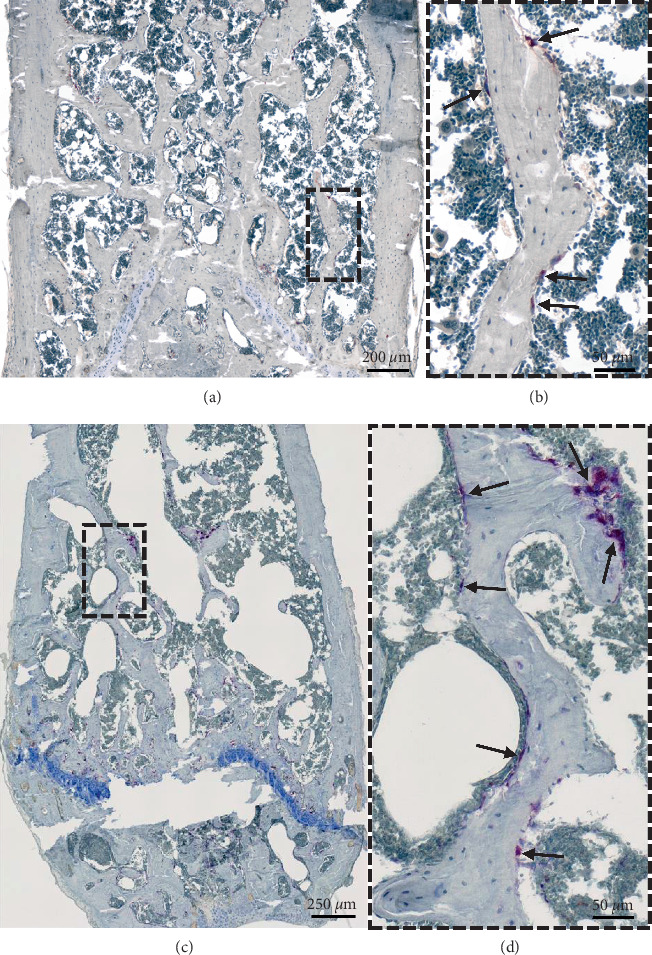
TRAP staining of femora injected with PBS (a, b) and MOPC315.BM.Luc cells (c, d) at day 21. (a–d) TRAP staining of the femoral condyle region to visualize osteoclast activity (purple) at day 21. (b, d) Detailed view of the rectangular area in (a) and (c) showing osteoclast activity on the surface of trabecular bone (purple, black arrows). Blue indicates nuclei (haematoxylin) counterstain.

Following euthanasia, femora were dissected and analyzed for characteristic signs of MM bone disease. Longitudinal sections of PMMA-embedded bones [14] were stained with TRAP and revealed high osteoclast activity in trabecular bone of MM-injected mice (Figures 2(c) and 2(d)) unlike PBS-injected bones (Figures 2(a) and 2(b)).

### 3.3. Osteolytic Bone Structural Changes Detected by Synchrotron Phase Contrast-Enhanced MicroCT

**Figure 3:**
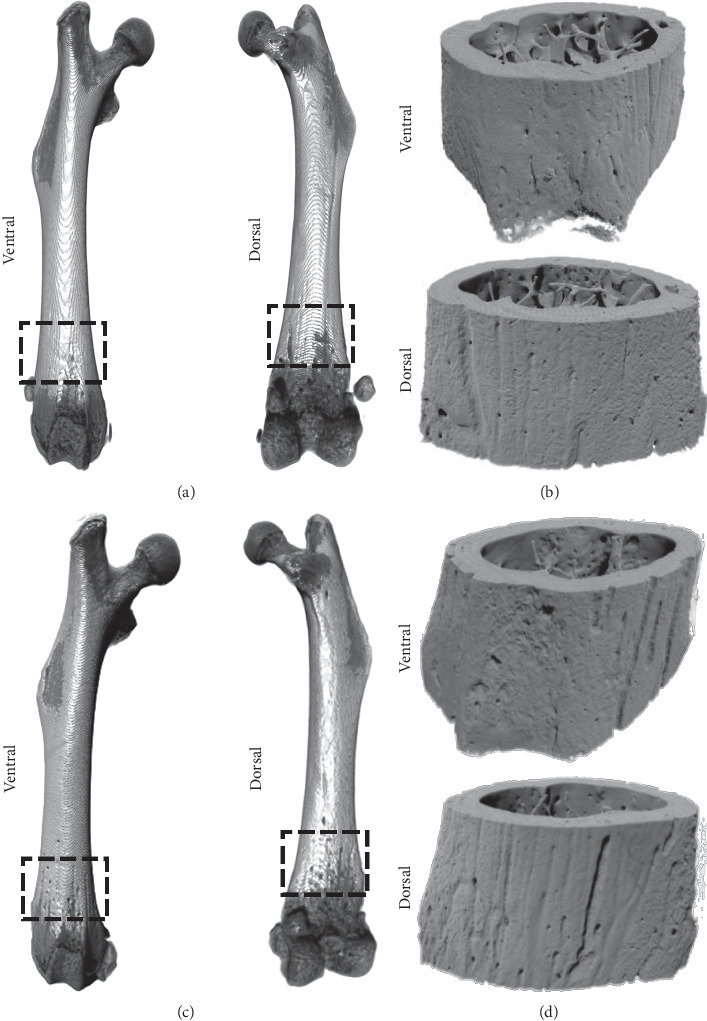
3D renderings of high-resolution microCT and PCE-CT for bone ultrastructural characterization of femora injected with PBS (a, b) versus MOPC315.BM.Luc cells (c, d) at day 21. (a, c) MicroCT images of ventral and dorsal views of one representative femur of PBS-injected (a) and MM-injected (c) bones. (b, d) Synchrotron PCE-CT 3D reconstructions of the same bones scanned by microCT reveal higher resolution detail in both ventral and dorsal regions just above the condyles.

**Figure 4:**
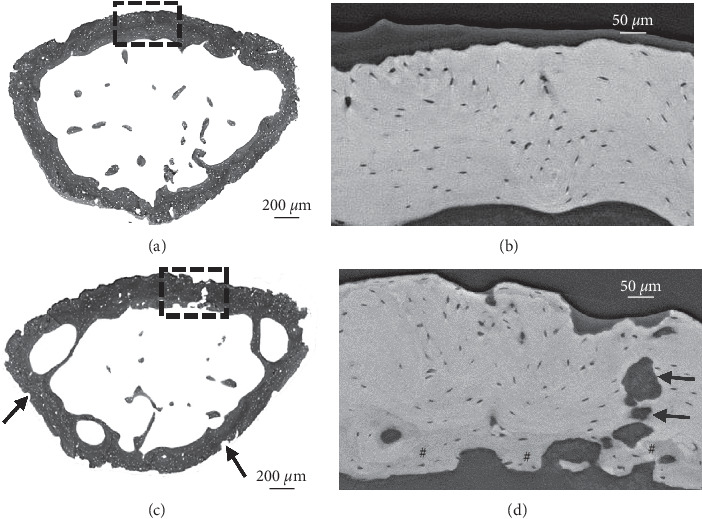
PCE-CT for bone ultrastructural characterization of femora injected with PBS (a, b) versus MOPC315.BM.Luc cells (c, d) at day 21. (a, c) PCE-CT images of cross sections of femur metaphysis. (c) Numerous sites of erosion (black arrows) and bone loss are indicated in the femur of the MM-injected bone. (b, d) High-magnification view of the rectangular area in (a) and (c). (d) Rectangular area of a local region in the cortex reveals multiple zones of intensive bone remodeling activity, with low-density new bone (darker grey, indicated with #) intermixed with mature bone sites (brighter grey), pocked with irregular-shaped “punched-out” lesions (black arrows).

**Figure 5:**
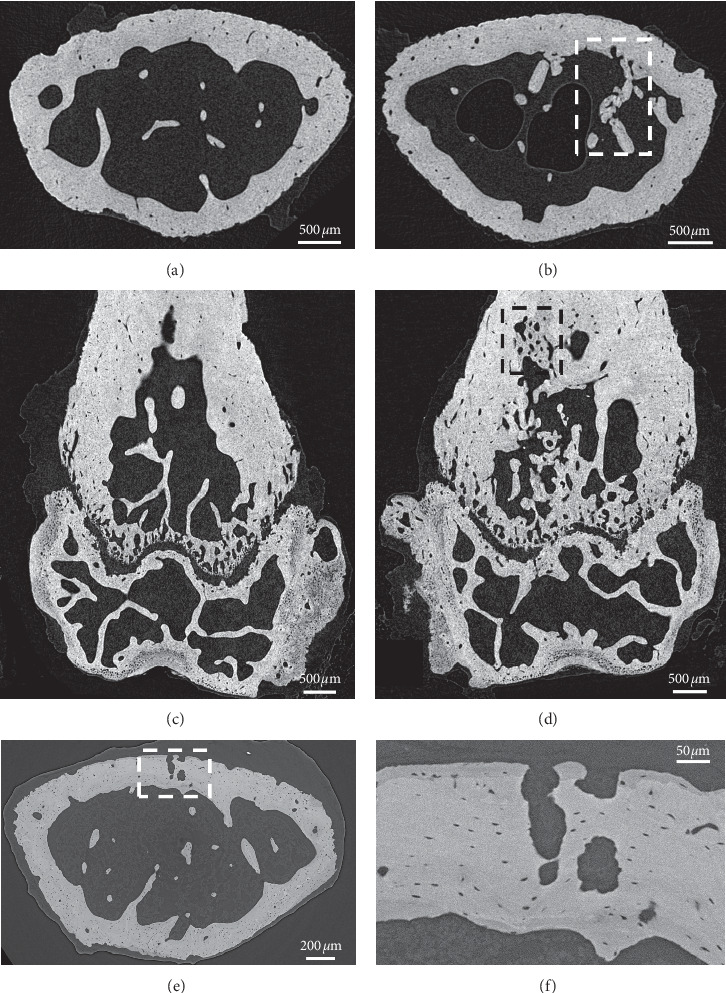
MicroCT and PCE-CT images of a control, left (noninjected), and a right femur injected with MOPC315.BM.Luc cells at day 15 after inoculation. Transversal cross sections of high-resolution microCT images of the left (a) and right (b) femur metaphysis demonstrate a typical region of disrupted trabeculae (highlighted by the rectangular area in (b)). Longitudinal, frontal cross sections of high-resolution microCT images of the left (c) and right (d) femora showing a region of high bone turnover and bone healing in the rectangular area in (d). (e) PCE-CT cross-sectional image of the same right femur metaphysis as shown in (b). (f) High-magnification view of the rectangular area in (e) reveals cavitation and irregular-shaped osteolytic lesions in the cortex.

Low-resolution microCT scans of MM-injected femora showed no visible signs of bone structure changes at days 7, 11, 15, and 21 after inoculation (Supplementary Figure 1). Similarly, reconstructed high-resolution microCT and PCE-CT images of a PBS-injected bone revealed no signs of osteolytic bone structure changes at 21 days after inoculation (Figures 3(a) and 3(b); Figures 4(a) and 4(b)). In contrast, in a MM-injected femur, high-resolution microCT scans displayed characteristic signs of intensive bone resorption at 21 days after inoculation (Figures 3(c) and 3(d); Figures 4(c) and 4(d)). High bone turnover and cavitation were observed on the outer bone surfaces, as indicated by multiple trenches and grooves (Figure 3(c)). Subtle microarchitecture differences were further revealed by region-of-interest imaging using PCE-CT (Figure 3(d)). Osteolytic bone structural changes were seen at the micrometer scale in cross sections of MM-injected bones (Figures 4(c) and 4(d)). The bone perimeter exhibited an overall jagged appearance with numerous sites of erosion (Figure 4(c), arrows), mixed with zones showing low-density newly formed bone (Figure 4(d), darker greyscale, indicated with #), juxtaposed with mature bone (Figure 4(d), brighter greyscale). The cortical bone was pocked with irregular-shaped “punched-out” lesions (Figure 4(d), black arrows). Bone ultrastructural characterization of femora at earlier time points at day 11 (data not shown) and day 15 (Figure 5) after inoculation showed a disruption of trabeculae (Figure 5(b)) and signs of bone healing with low-density newly formed bone in the injected area (Figure 5(d)), different from what was observed in the contralateral left femur (Figures 5(a) and 5(c)). PCE-CT images revealed irregular-shaped osteolytic lesions already at day 15 (Figures 5(e) and 5(f)).

### 3.4. Mineralized and Nonmineralized Matrix Changes in the Osteocyte Lacunar-Canalicular Network

**Figure 6:**
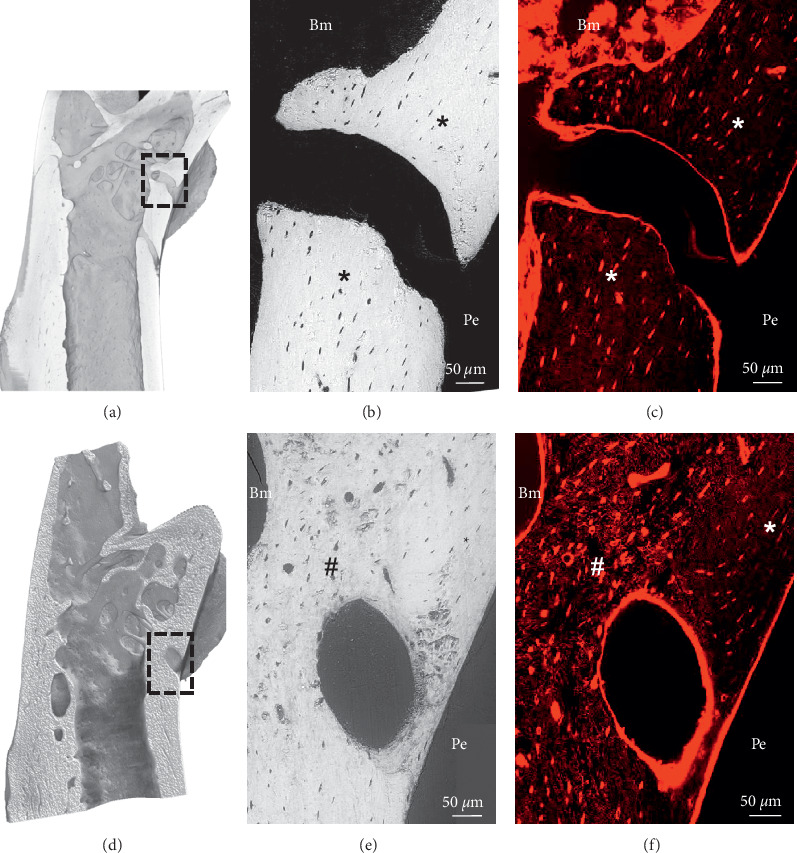
Electron and fluorescence confocal laser scanning microscopy for bone ultrastructural characterization of femora injected with PBS (a–c) and MOPC315.BM.Luc cells (d–f) at day 21. (a, d) 3D renderings of microCT scans of embedded proximal femora showing large cavities corresponding to blood vessels in the cortical bone. (b, c) Detailed views of the region indicated by the rectangle in (a). (b) BSE image shows intact osteocyte lacunae (indicated with ∗). (c) CLSM imaging of the rhodamine-stained sample reveals only well-organized OLCN (indicated with ∗). (e, f) Detailed views of the region indicated by the rectangle in (d). (e) BSE image reveals osteocyte cross sections and local variations in mineral content, based on signal from the mineralized components of the matrix. (f) CLSM imaging of the rhodamine-stained sample reveals nonmineralized regions within cortical bone, including osteocyte lacunae and canaliculi network. Correlative BSE and CLSC imaging show flat osteocyte lacunae organized (indicated with ∗) in a lamellar structure on the periosteal region (Pe) and larger, irregular-shaped lacunae within a disorganized (indicated with #) canaliculi network architecture in the proximity of the bone marrow (Bm).

3D renderings of lab-CT revealed large cavities in the cortical bone at the proximal femur of a PBS (Figure 6(a)) and a MOPC315.BM.Luc cell-injected mouse at day 21 (Figure 6(d)), corresponding to transcortical blood vessels connecting the outer surface with the bone marrow (Movie S1 of a MM-injected mouse), as recently demonstrated [19]. Correlative backscattered electron imaging (BSE; Figures 6(b) and 6(e)) and confocal laser scanning microscopy (CLSM; Figures 6(c) and 6(f)) allowed direct comparison of mineralized and nonmineralized matrix changes in the cortical bone. BSE imaging of MM-injected bones showed irregular small and larger cavities distributed throughout the cortical bone of the MM-injected bone, corresponding to intact (indicated with ∗) and altered (indicated with #) osteocyte lacunae as well as local changes in the degree of mineralization (Figure 6(e)). PBS-injected bones revealed only intact (indicated with ∗) osteocyte lacunae (Figure 6(b)). The OLCN was visualized by CLSM following staining of the bone cavities with rhodamine [16], which binds to nonmineralized surfaces (Figures 6(c) and 6(f)). In PBS-injected femora, only well-organized (indicated with ∗) OLCN was observed (Figure 6(c) and Movie S2 and S3). Large and irregular-shaped osteocyte lacunae were seen within a disorganized OLCN architecture in MM-injected bones (Figure 6(f), indicated with #; Movie S4). In contrast, smaller flat oval-shaped osteocytes with a well-organized OLCN arranged in a lamellar structure were visible in the periosteal region (Figure 6(f), indicated with ∗). CLSM imaging of the rhodamine-stained sample showed in a second region of interest a similar mixture of disorganized and organized OLCN next to each other (Supplementary Figure S2).

**Figure 7:**
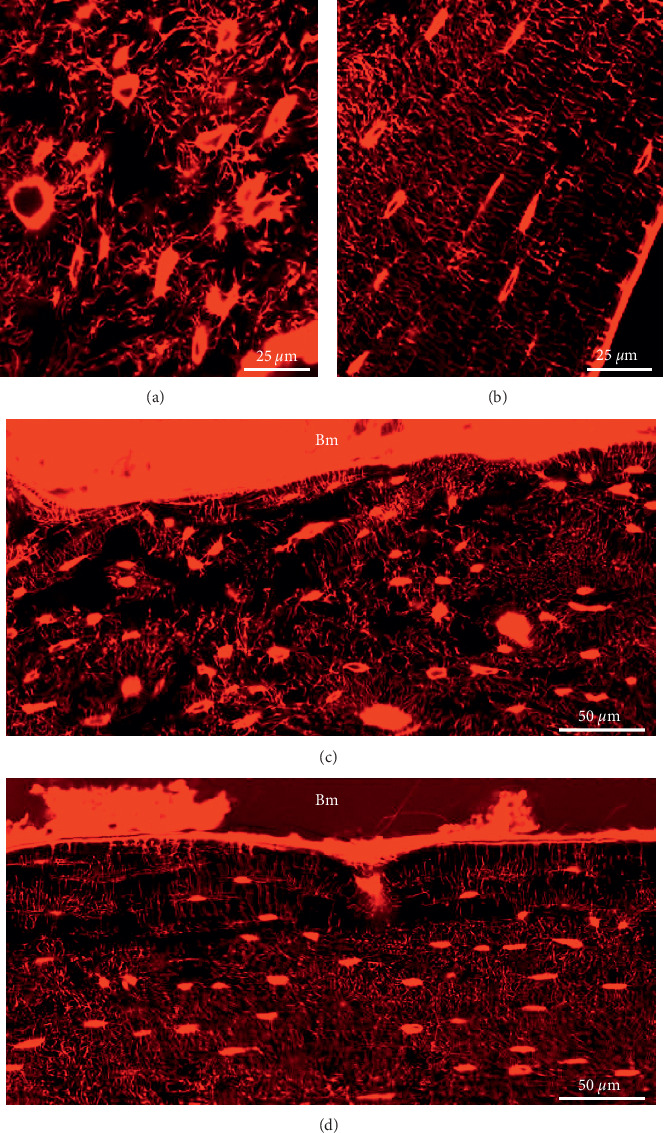
High-magnification images of OLCN stained with rhodamine and visualized with fluorescence confocal laser scanning microscopy. MM-injected femur with (a) larger, irregular-shaped osteocyte lacunae within a disorganized canaliculi network architecture in proximity to the bone marrow (indicated with # in Figure 3(f)) and (b) flat osteocyte lacunae organized in a lamellar structure on the periosteal side (indicated with ∗ in Figure 3(f)). High-magnification image of the connection of the osteocyte canaliculi network to the bone marrow on the endosteal surface of (c) a MM-injected femur with a disrupted network and (d) a healthy control mouse femur illustrating an organized network in lamellae parallel to the bone surface and canaliculi perpendicular to them.

High-magnification images of the osteocyte morphology in the MM-injected femur (Figure 6(f)) showed larger, irregular-shaped osteocyte lacunae within a disorganized canaliculi network architecture (Figure 7(a)) next to flat osteocyte lacunae organized in a lamellar structure (Figure 7(b)). Quantification of two regions of interest of the osteocyte lacunae volume and canalicular density in the MM-injected femur revealed that disorganized regions had a greater lacunae volume (365 *μ*m^3^) and sparser canalicular density (0.137 ± 0.11 *μ*m/*μ*m^3^) compared to regions in the PBS-injected bone (249 *μ*m^3^; 0.155 ± 0.09 *μ*m/*μ*m^3^; respectively).

Osteocytes communicate with other osteocytes and with cells on the bone periosteal or endosteal surface, such as lining cells, stromal cells, osteoblasts, and/or osteoclasts, through an intricate canaliculi network [20, 21]. The connection of the OLCN to the bone marrow on the endosteal surface revealed a disrupted network in a MM-injected femur (Figure 7(c)). In a PBS-injected femur, the network was organized in lamellae parallel to the bone surface and canaliculi perpendicular to it (Figure 7(d)).

## 4. Discussion

Our results demonstrate early MM bone disease in adult 26-week-old BALB/c mice using MOPC315.BM.Luc cells. The rationale for using 26-week-old mice was that it has been documented that these mice reach a state of skeletal maturity [9, 22, 23]. While housing 26-week-old mice is more expensive, time-consuming, and tricky as compared to younger mice, it ascertains the relevance of our model to study MM bone effects in skeletally mature animals, although we are aware that there is value in studies using younger aged animals. Our purpose here was to develop a relevant model resembling as best as is practical adult human populations, although mice do not have the same osteonal bones as humans do. Human MM disease develops in aging populations with a medium age of 69 at first diagnosis. It has been shown that age-related changes in the BALB/c skeleton mimic those in aging humans [24]. The moment of inertia increases while the cortical area is maintained during aging in mice and this is in line with endosteal and periosteal expansion seen in human long bones [24]. Also, the decreased trabecular bone volume fraction observed in aged mice is comparable to the trabecular bone loss in elderly humans [24]. Further, the decreased fracture energy detected in aged mice is relevant to the decline in toughness seen in studies on human cortical bone [24]. In this respect, younger mice (6 to 16 weeks old) would not resemble the bone turnover of aging humans. The animal age we chose makes it feasible both to study all stages of rapid MM bone disease and to test new treatment protocols in the future.

Our newly developed syngeneic model is locally confined and shows potential for future characterization of different stages of the disease down to the ultrastructural level. It differs substantially from previously established syngeneic models such as the 5TMM model, where two distinctly different MM cell lines are used: (i) the moderate 5T2MM cells or (ii) the more aggressive 5T33MM cells. In both cases, MM cells must be injected intravenously (i.v.) into young 6-week-old mice with osteolytic lesions appearing only after 11∼16 weeks, when mice showed end-stage disease and tumors [25, 26]. While useful for some studies, lesions in these models are not primarily localized to one particular bone [26], as is the case in our model. In the aggressive 5T33MM model as in the MOPC315.BM.Luc model after i.v. injection [6], tumors grow in multiple organs including nonhematopoietic tissue. Mice often succumb to the disease without necessarily showing signs of lesions in long bones. All these make it difficult to track specific stages of MM bone disease development, a challenge that our MOPC315.BM.Luc model circumvents.

Our newly established model in skeletally mature bones of BALB/c mice provides a unique tool for studying MM bone disease in different stages of disease progression, for at least 3 weeks after MM cell injection. The results shown here provide first insights into quantitative data of the BLI of mice 3, 7, 11, 15, and 21 days after i.f. MOPC315.BM.Luc cell inoculation, and qualitative data based on high-resolution microCT and PCE-CT and confocal and electron microscopy, as proof of concept for the detection of subtle ultrastructural changes in the extracellular matrix of MM-affected bones. The model is strengthened by its confinement to one particular bone, thus minimizing animal suffering and improving localization, which is essential for using high-resolution characterization methods in restricted small regions.

Detection of increased osteoclast activity evidenced by TRAP staining indicates active osteolytic disease in MOPC315.BM.Luc-injected mice. In future studies, our model can be used for the detection of osteoblast suppression, which is a hallmark of myeloma bone disease. Expression of markers for osteogenic differentiation such as the osteocytic marker sclerostin, dentin matrix protein 1, and fibroblast growth factor 23, as well as the osteogenic markers alkaline phosphatase, bone *γ*-carboxylglutamic acid-containing protein, and osteopontin, could be compared between PBS- and MOPC315.BM.Luc-injected femora by immunohistochemical analysis.

Contemporary research of bone tissue routinely employs microCT with resolutions down to 5∼10 micrometers. High-resolution microCT down to and below one micrometer is challenging but is important to identify and characterize the length scale of osteocytes. Although increasingly studied by high-resolution microCT, synchrotron-based PCE-CT scans reveal structural attributes with target resolutions below 100 nm, in realistic millimeter-sized samples. Thus, combining high-resolution microCT with synchrotron PCE-CT, it is possible to detect osteolytic bone structural changes in the sub-micrometer scale. With PCE-CT, all bone compartments may be visualized in 3D, including subtle differences in the degree of mineralization of the bones [27]. In this manner, important density differences and gradients are revealed by synchrotron beamlines employing advanced iterative methods of ptychography, and they are well able to spatially map small changes below <100 nm resolution, within millimeter-sized samples of tooth dentin [28]. Future studies of MM-affected bones will thus provide insights into the very early stages of MM bone disease.

We detected increased osteoclastic activity on the bone interface. Furthermore, complementary analyses using BSE and CLSM allowed the detection of changes in the bone ultrastructure, down to single osteocyte lacunae and canaliculi network in the nanometer scale revealing spatial alterations in the network architecture. Large and irregular-shaped osteocyte lacunae detected within the disorganized OLCN suggest locally altered bone remodeling, probably indicating increased bone destruction in the perilacunar space in MM-injected femora. In addition to the altered size and shape of osteocyte lacunae, the disrupted canaliculi network architecture is indicative of a bone pathology in our MM bone disease model. Indeed, it is well known that changes in the bone ultrastructure result in pathological conditions, such as osteoporosis. A recent study on a murine osteoporosis model investigates the influence of lacunar-canalicular permeability and vascular porosity on the fluid flow magnitude and shows that this was significantly reduced in an osteoporotic condition as modeled with ovariectomized rats [29]. This reduction in fluid flow could impair the mechanosensory function of osteocytes and lead to age-related bone loss in postmenopausal osteoporosis [29].

Our model thus paves the way for combining complementary modalities for characterizing the extracellular matrix ultrastructure, with great potential for a deeper understanding of the pathological changes at all stages of MM disease. Such information is critical for the development of next-generation treatment approaches.

In summary, we developed an in vivo, confined MM disease model, in skeletally mature (26 weeks old) BALB/c mice affecting femora of mice. This model will allow future combined biomaterial science and pharmacological investigations for early detection and treatment of MM.

## Data Availability

The data used to support the findings of this study are included within the article.

## Supplementary Materials

Supplementary Materials Supplementary Figure S1: Low-resolution lab-CT images of ventral and dorsal views of a representative femur of MM-injected bones at day (A) 7, (B) 11, (C) 15, and (D) 21, respectively. Supplementary Figure 2: Electron and confocal microscopy for bone ultrastructural characterization of femora injected with MOPC315.BM.Luc cells at day 21. (A) 3D rendering of a microCT scan, (B) BSE image of the proximal femur, and (C) CLSM imaging of the rhodamine-stained sample showing a detailed view of the region indicated by the rectangle in B. Bv indicates a transcortical blood vessel, Bm indicates bone marrow, and Pe indicates periosteal side. Movie S1: High-resolution lab-CT (voxel size 2.5 *μ*m) movie of a PMMA-embedded proximal femur 21 days after injection of MM cells. Movie shows that the large cavities in the cortical bone are channels that connect the outer surface with the bone marrow. Movie corresponds to Figure 6(d). Movie S2: OLCN of a PBS-injected femur stained with rhodamine and visualized with fluorescence confocal laser scanning microscopy. Still image corresponds to Figure 6(c). Magnification 40x, oil objective, 0.75 zoom, 6 tiles, 60 *μ*m total depth at 0.4 *μ*m step size. Movie S3: OLCN of a PBS-injected femur stained with rhodamine and visualized with fluorescence confocal laser scanning microscopy. Detail view of a region below the region indicated by the rectangle in Figure 6(a). Magnification 40x, oil objective, 0.75 zoom, 6 tiles, 60 *μ*m total depth at 0.4 *μ*m step size. Movie S4: OLCN of a MM-injected femur stained with rhodamine and visualized with fluorescence confocal laser scanning microscopy. Still image corresponds to Figure 6(f). Magnification 40x, oil objective, 0.75 zoom, 6 tiles, 60 *μ*m total depth at 0.4 *μ*m step size.
