# Supplementary material for: An Early Myeloma Bone Disease Model in Skeletally Mature Mice as a Platform for Biomaterial Characterization of the Extracellular Matrix
Source: J Oncol. 2020 Jun 27;2020:3985315. doi: 10.1155/2020/3985315 (PMC7336213; doi:10.1155/2020/3985315)
Supplement: Supplementary Materials — Supplementary Figure S1: Low-resolution lab-CT images of ventral and dorsal views of a representative femur of MM-injected bones at day (A) 7, (B) 11, (C) 15, and (D) 21, respectively. Supplementary Figure 2: Electron and confocal microscopy for bone ultrastructural characterization of femora injected with MOPC315.BM.Luc cells at day 21. (A) 3D rendering of a microCT scan, (B) BSE image of the proximal femur, and (C) CLSM imaging of the rhodamine-stained sample showing a detailed view of the region indicated by the rectangle in B. Bv indicates a transcortical blood vessel, Bm indicates bone marrow, and Pe indicates periosteal side. Movie S1: High-resolution lab-CT (voxel size 2.5 μm) movie of a PMMA-embedded proximal femur 21 days after injection of MM cells. Movie shows that the large cavities in the cortical bone are channels that connect the outer surface with the bone marrow. Movie corresponds to Figure 6(d). Movie S2: OLCN of a PBS-injected femur stained with rhodamine and visualized with fluorescence confocal laser scanning microscopy. Still image corresponds to Figure 6(c). Magnification 40x, oil objective, 0.75 zoom, 6 tiles, 60 μm total depth at 0.4 μm step size. Movie S3: OLCN of a PBS-injected femur stained with rhodamine and visualized with fluorescence confocal laser scanning microscopy. Detail view of a region below the region indicated by the rectangle in Figure 6(a). Magnification 40x, oil objective, 0.75 zoom, 6 tiles, 60 μm total depth at 0.4 μm step size. Movie S4: OLCN of a MM-injected femur stained with rhodamine and visualized with fluorescence confocal laser scanning microscopy. Still image corresponds to Figure 6(f). Magnification 40x, oil objective, 0.75 zoom, 6 tiles, 60 μm total depth at 0.4 μm step size. [file 3985315.f1.zip › Supplementary data.docx]

**An early myeloma bone disease model in skeletally mature mice as a platform for biomaterial characterization of the extracellular matrix**

Fani Ziouti, Ana Prates, Inés Moreno-Jiménez, Alexander Rack, Bjarne Bogen, Amaia Cipitria, Paul Zaslansky, Franziska Jundt

Supplementary Figure S1

Supplementary Figure S2

Supplementary Movie S1

Supplementary Movie S2

Supplementary Movie S3

Supplementary Movie S4

A

ventral

50 μm

C

B


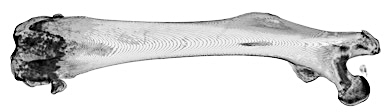

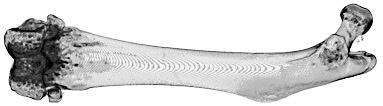


dorsal


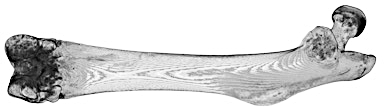

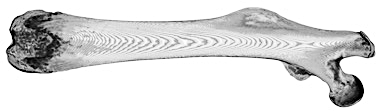

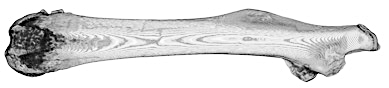

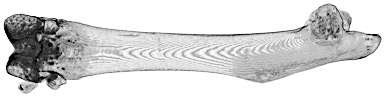

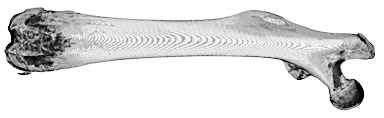

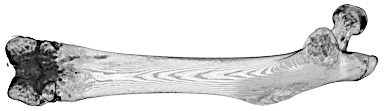


ventral

ventral

ventral

dorsal

dorsal

dorsal

D

Supplementary Figure S1: Low-resolution lab-CT images of ventral and dorsal views of a representative femur of MM injected bones at day (A) 7, (B) 11, (C) 15 and (D) 21, respectively.


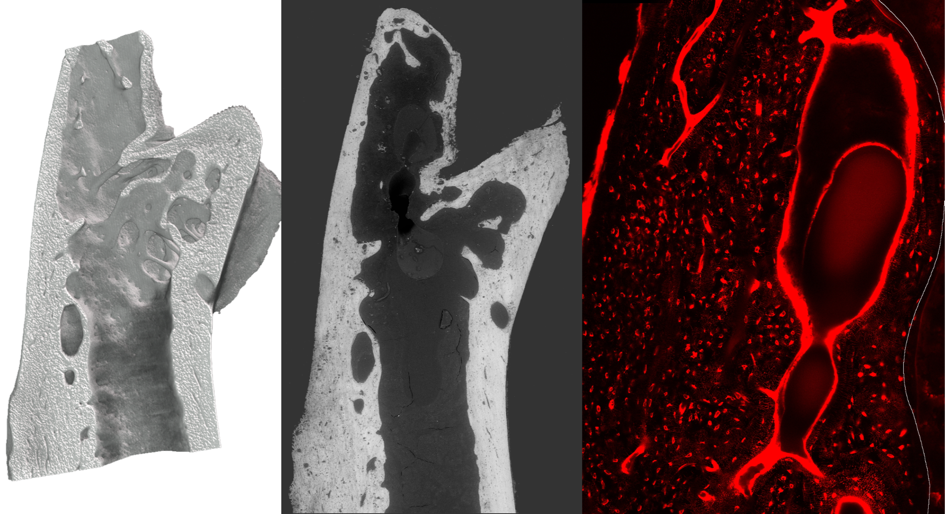


500 μm

100 μm

A

B

C

Pe

Bv

Bm

Bm

Supplementary Fig. 2: Electron and confocal microscopy for bone ultrastructural characterization of femora injected with MOPC315.BM.Luc cells at day 21. (A) 3D rendering of a microCT scan, (B) BSE image of the proximal femur and (C) CLSM imaging of the rhodamine stained sample showing a detailed view of the region indicated by the rectangle in B. Bv indicates a transcortical blood vessel, Bm indicates bone marrow and Pe indicates periosteal side.

**
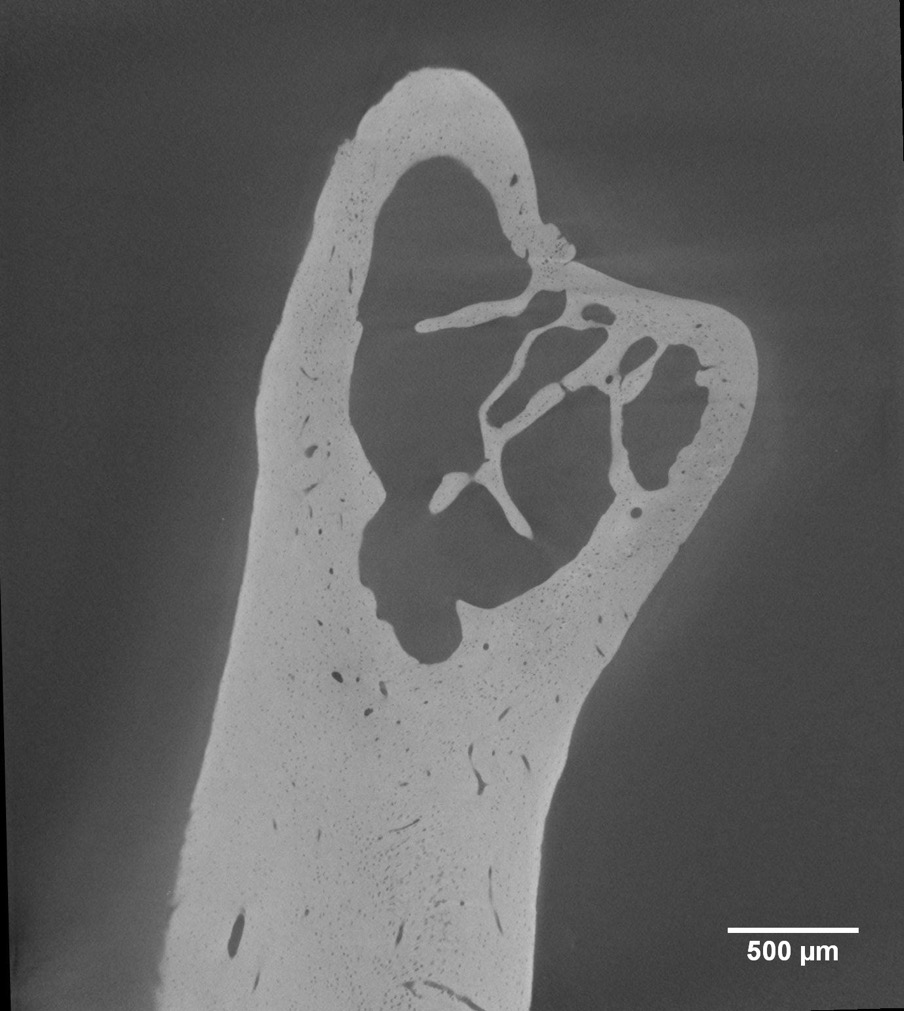
**

Movie S1: High-resolution lab-CT (voxel size 2.5 μm) movie of a PMMA-embedded proximal femur 21 days after injection of MM cells. Movie shows that the large cavities in the cortical bone are channels that connect the outer surface with the bone marrow. Movie corresponds to Fig. 6D.


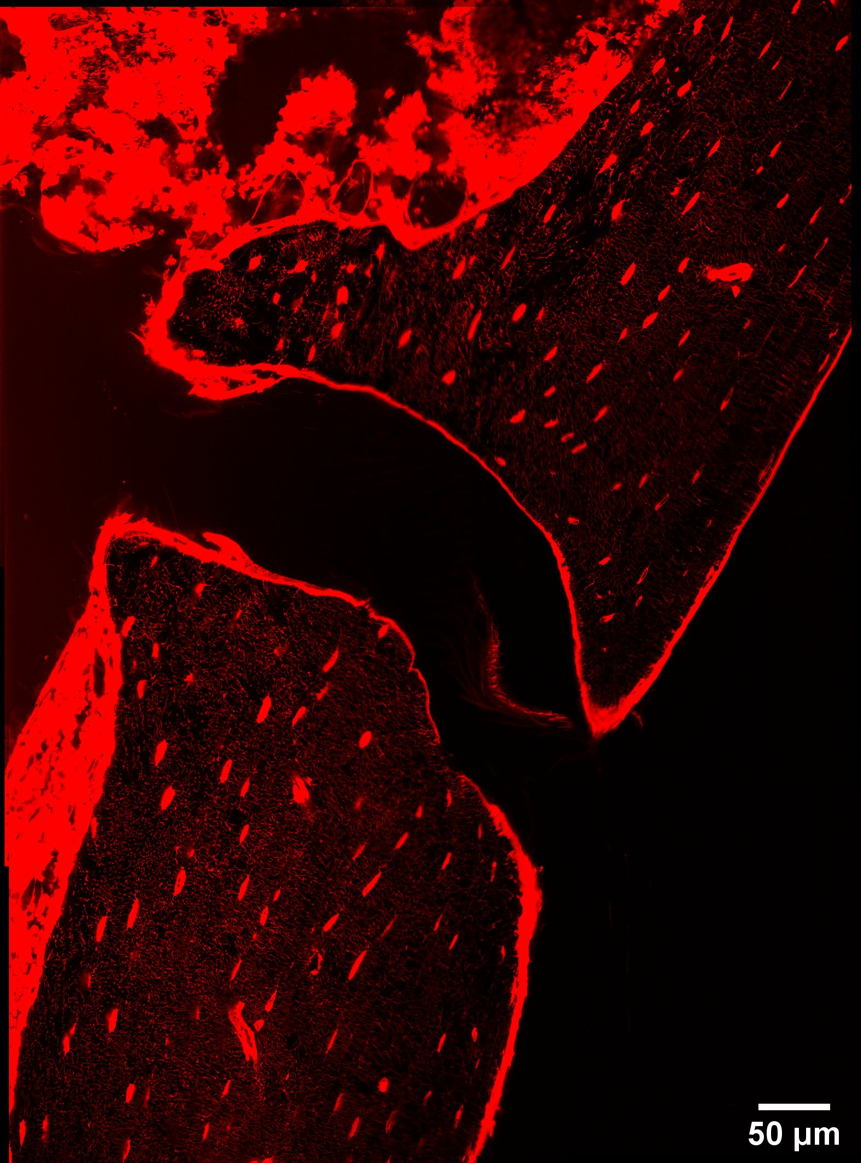


Movie S2: OLCN of a PBS injected femur stained with rhodamine and visualized with fluorescence confocal laser scanning microscopy. Still image corresponds to Fig. 6C. Magnification 40x, oil objective, 0.75 zoom, 6 tiles, 60 μm total depth at 0.4 μm step size.


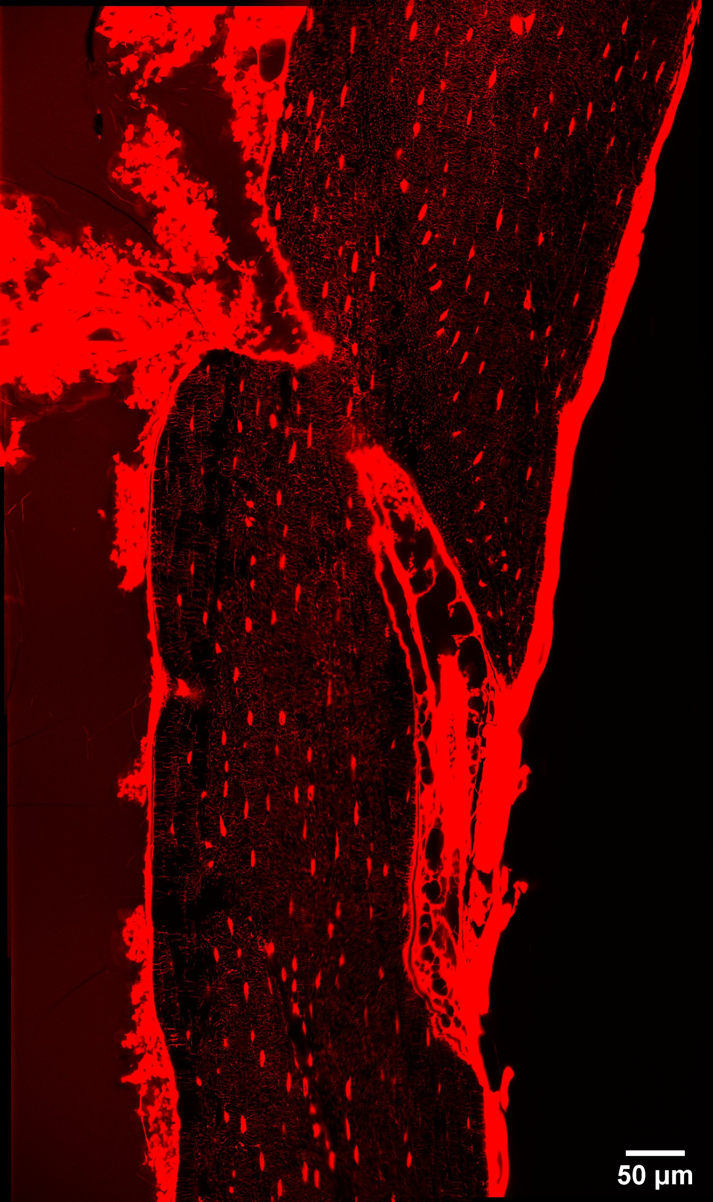


Movie S3: OLCN of a PBS injected femur stained with rhodamine and visualized with fluorescence confocal laser scanning microscopy. Detail view of a region below the region indicated by the rectangle in Fig. 6A. Magnification 40x, oil objective, 0.75 zoom, 6 tiles, 60 μm total depth at 0.4 μm step size.


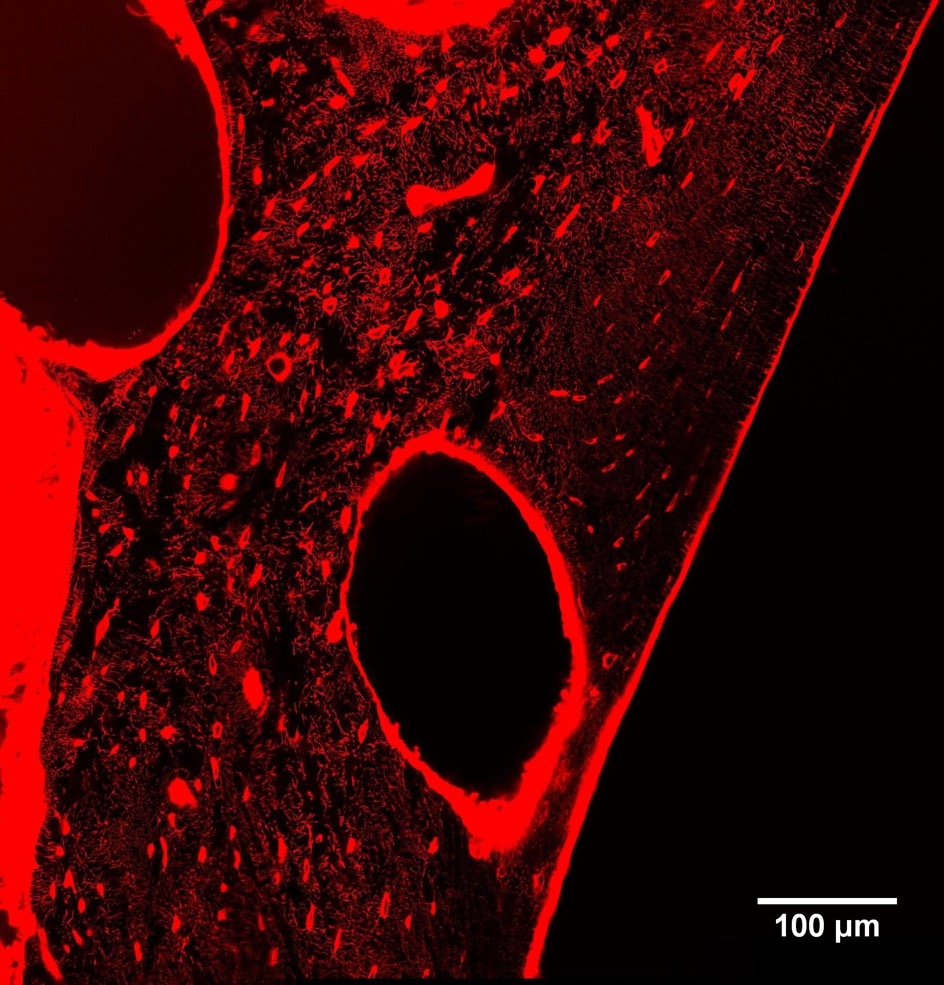


Movie S4: OLCN of a MM injected femur stained with rhodamine and visualized with fluorescence confocal laser scanning microscopy. Still image corresponds to Fig. 6F. Magnification 40x, oil objective, 0.75 zoom, 6 tiles, 60 μm total depth at 0.4 μm step size.
